# Supplementary figures and images for: Protein kinase Cδ is essential for the IgG response against T-cell-independent type 2 antigens and commensal bacteria
Source: eLife. 2021 Oct 25;10:e72116. doi: 10.7554/eLife.72116 (PMC8610492; doi:10.7554/eLife.72116)

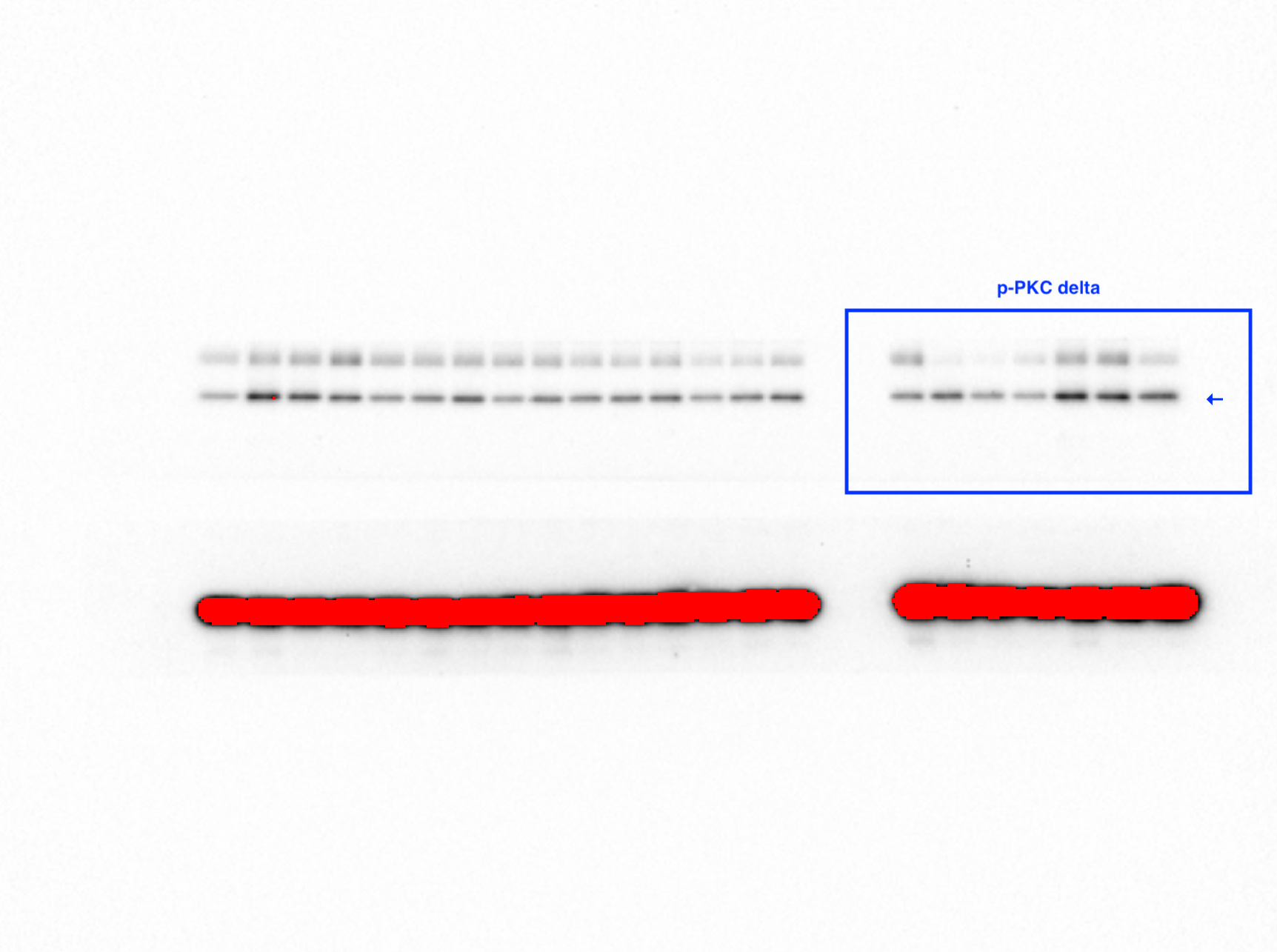

Supplement: Figure 2—source data 2. [file elife-72116-fig2-data2.tif]

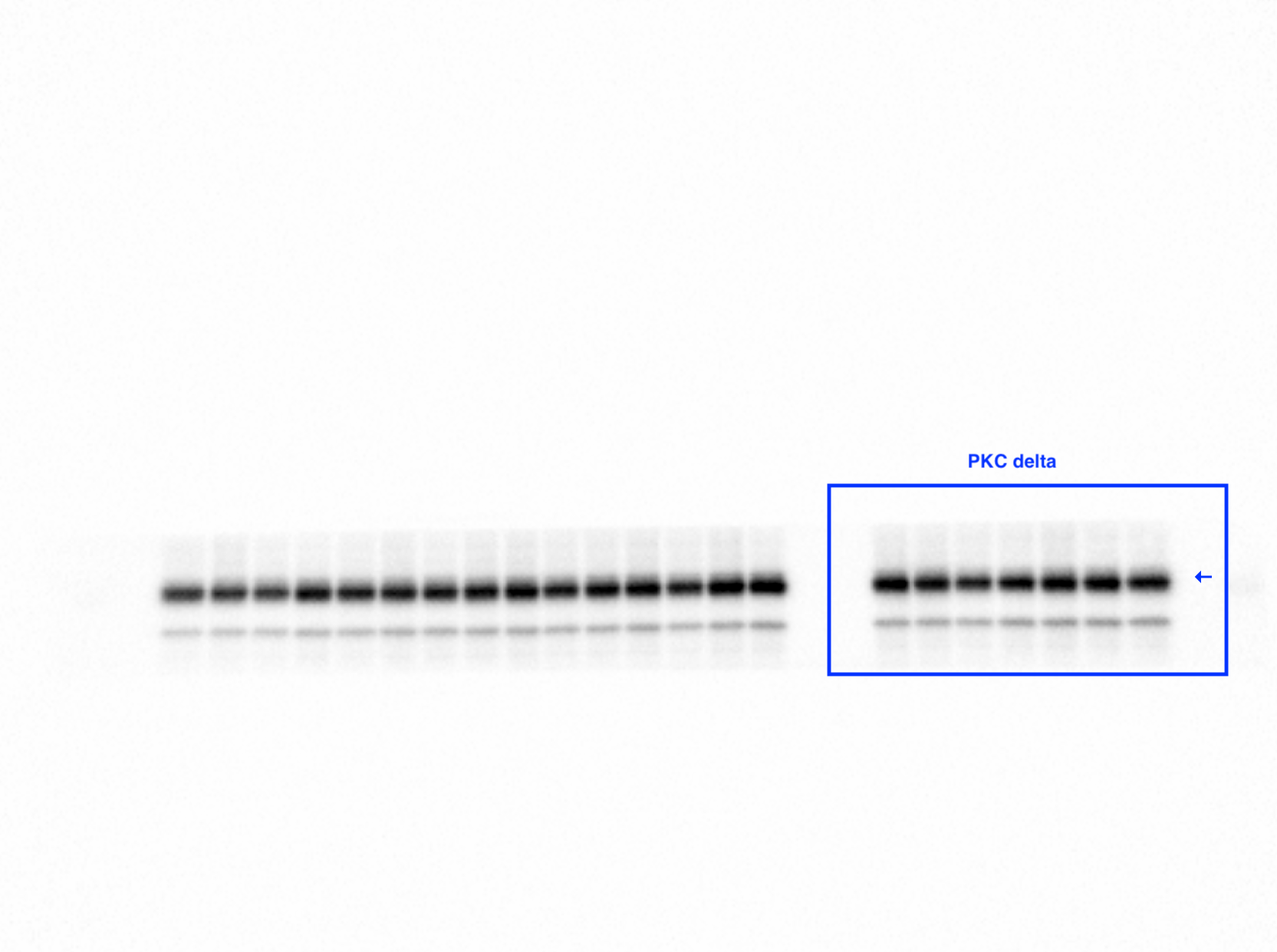

Supplement: Figure 2—source data 3. [file elife-72116-fig2-data3.tif]

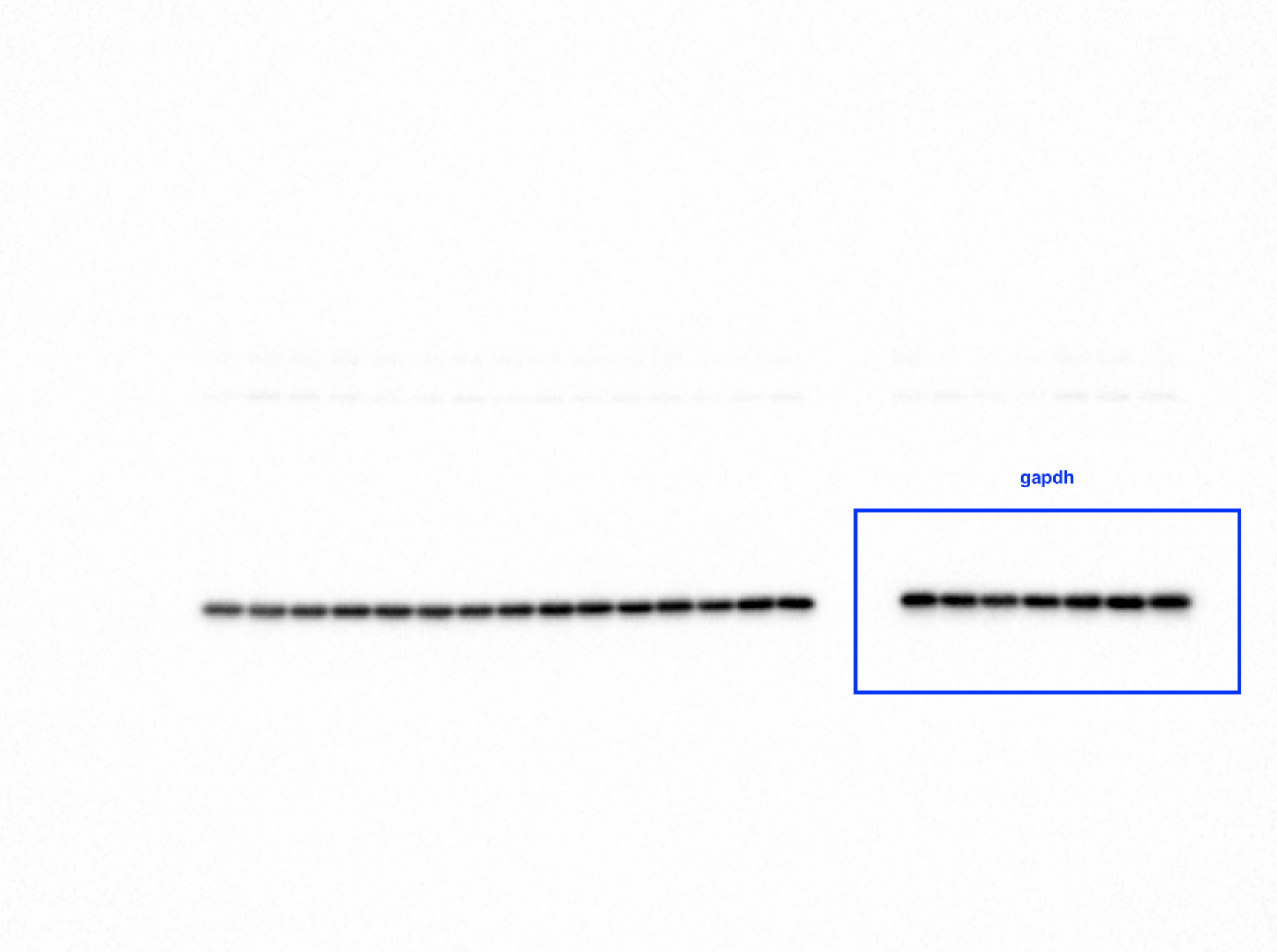

Supplement: Figure 2—source data 4. [file elife-72116-fig2-data4.tif]

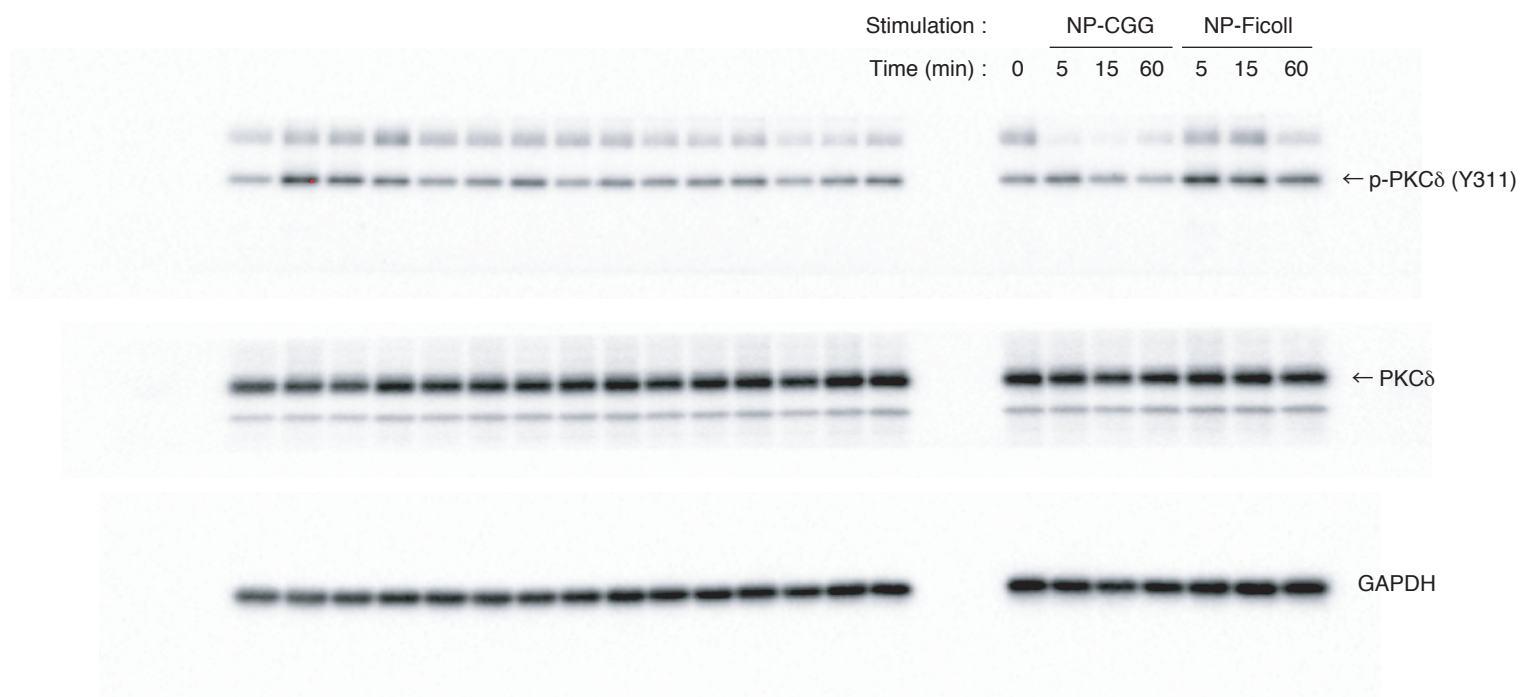

Supplement: Figure 2—source data 5. [file elife-72116-fig2-data5.pdf]

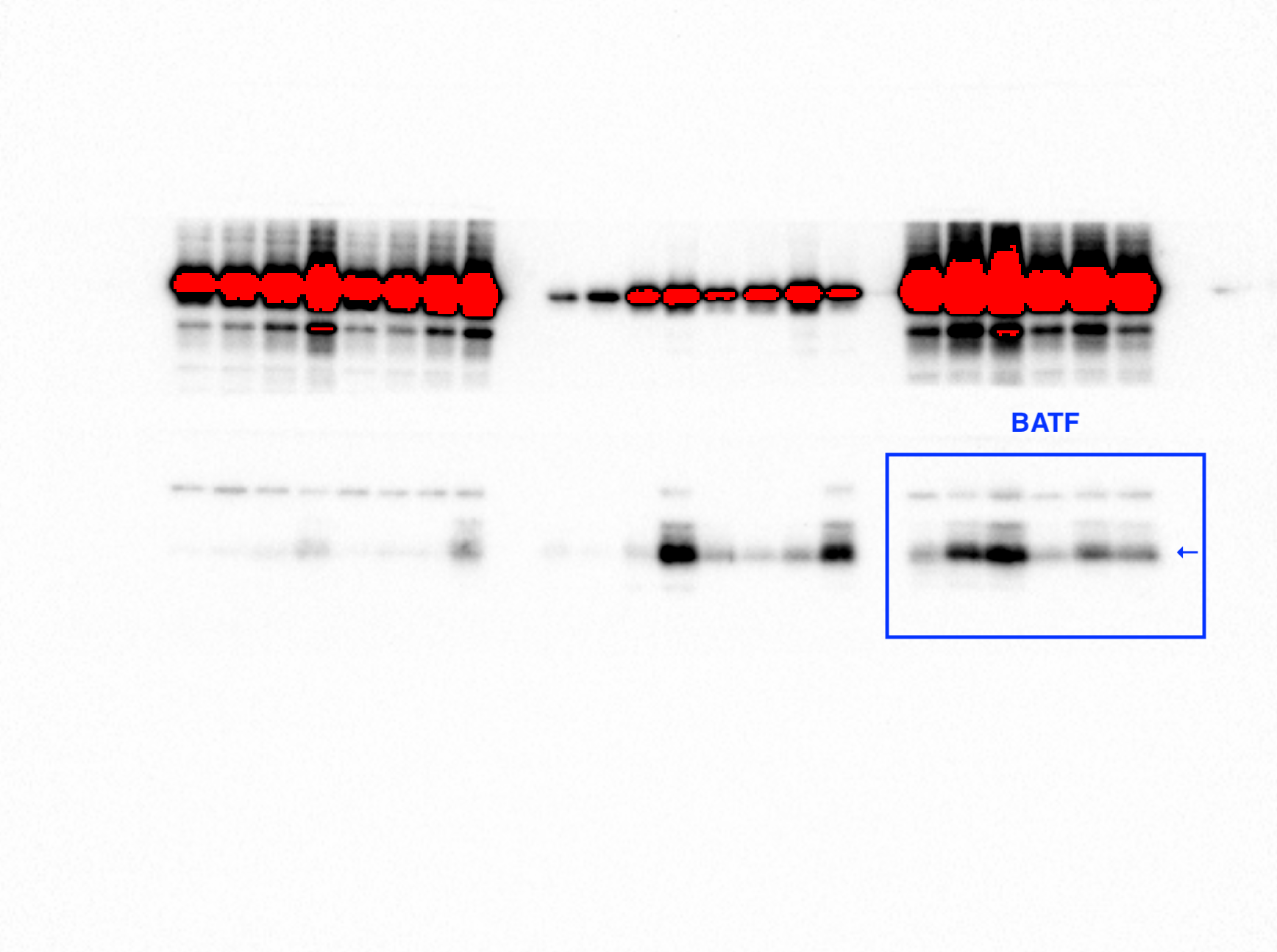

Supplement: Figure 5—source data 2. [file elife-72116-fig5-data2.tif]

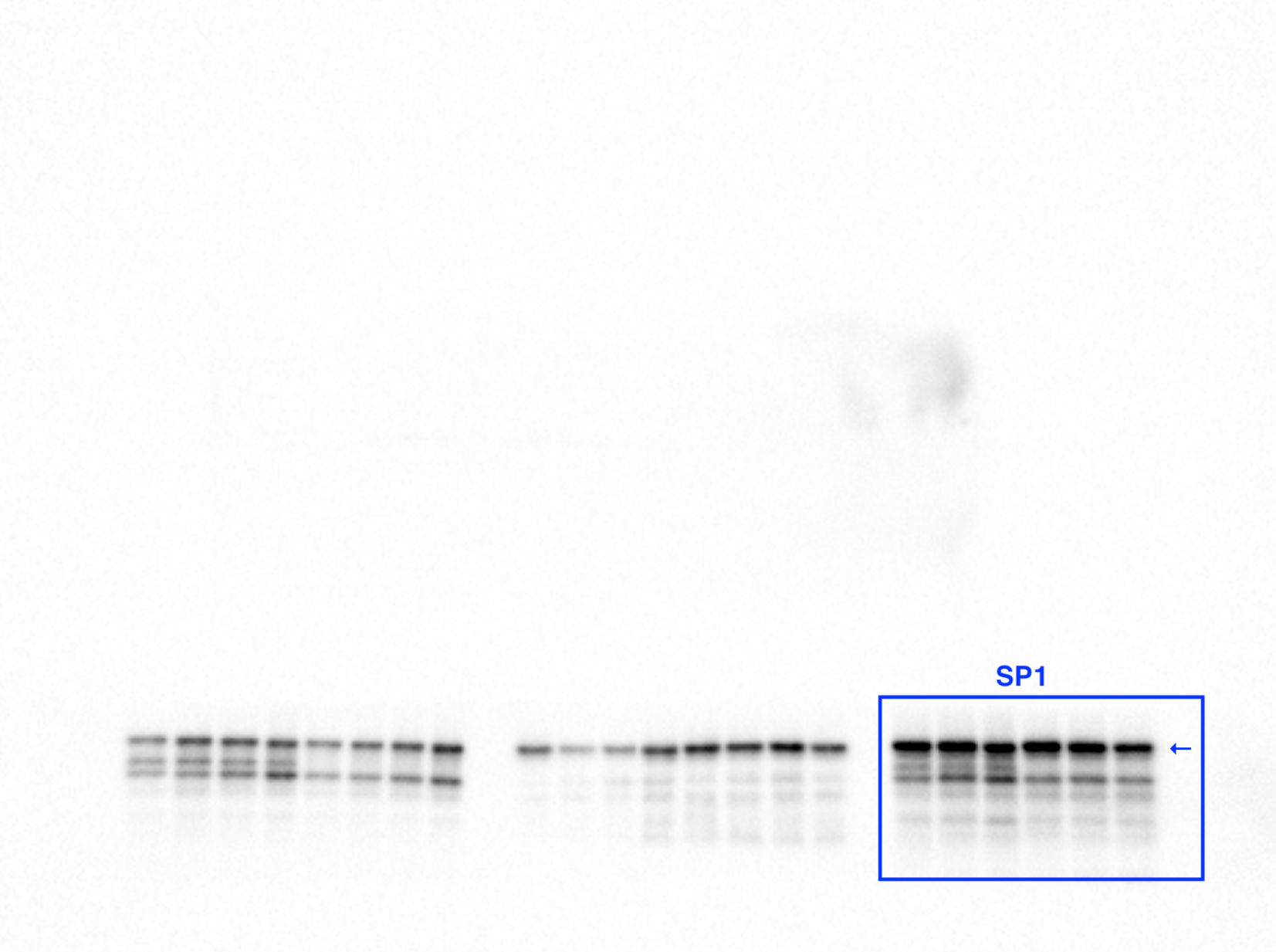

Supplement: Figure 5—source data 3. [file elife-72116-fig5-data3.tif]

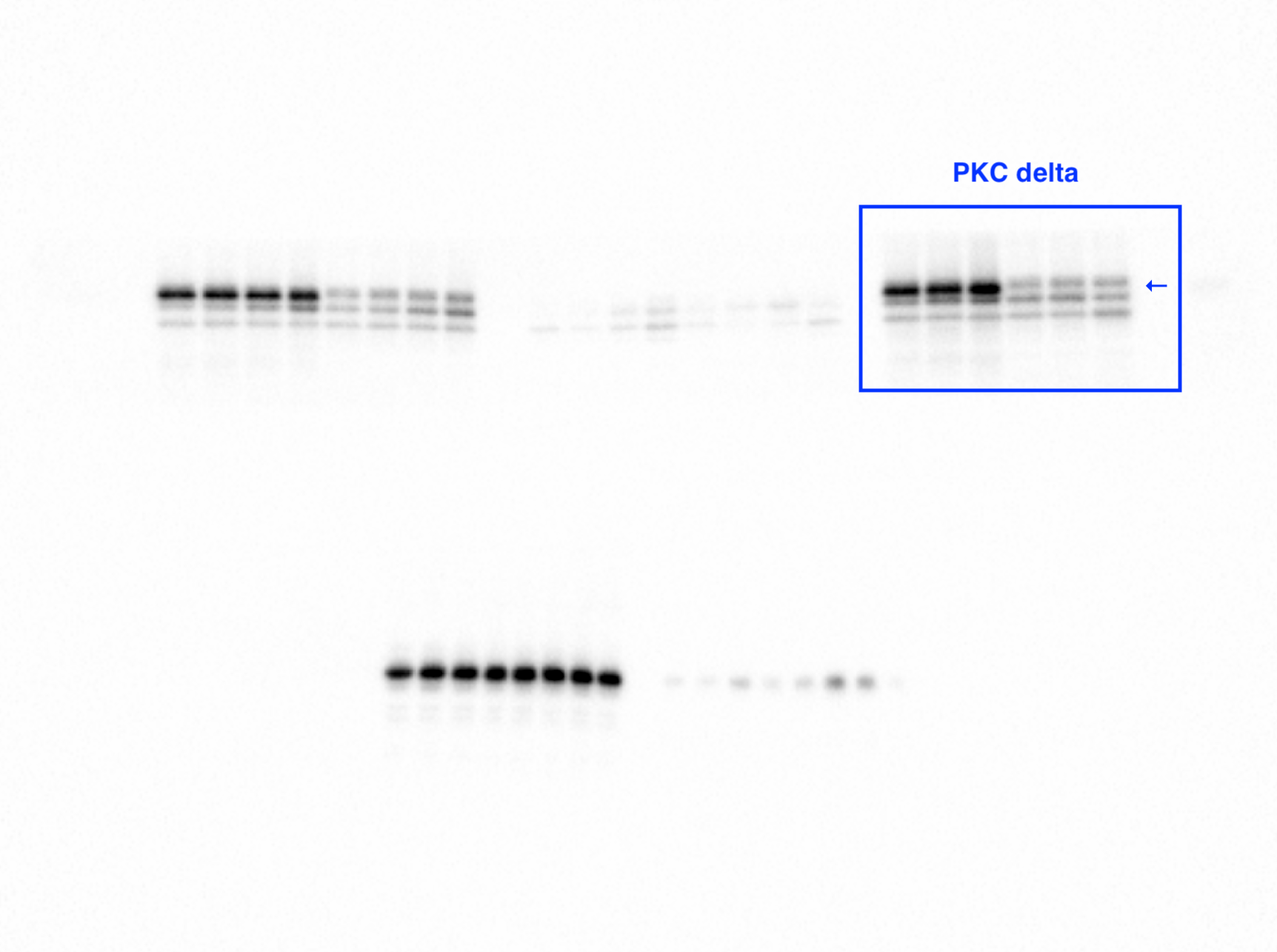

Supplement: Figure 5—source data 4. [file elife-72116-fig5-data4.tif]

|               | <i>Prkcd</i> <sup>+/+</sup> |    |    | <i>Prkcd</i> <sup>-/-</sup> |    |    |
|---------------|-----------------------------|----|----|-----------------------------|----|----|
| Time (hour) : | 0                           | 24 | 48 | 0                           | 24 | 48 |

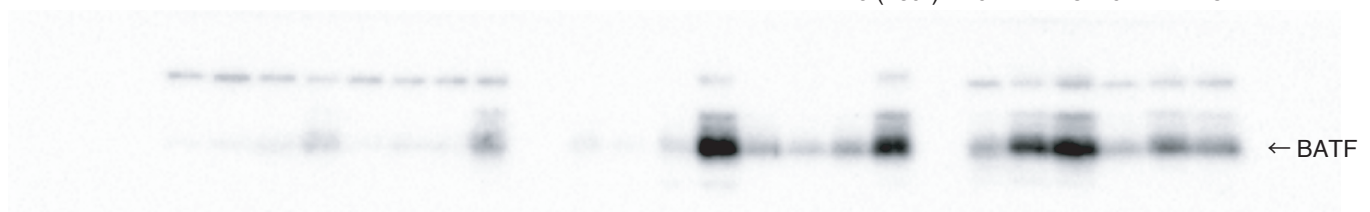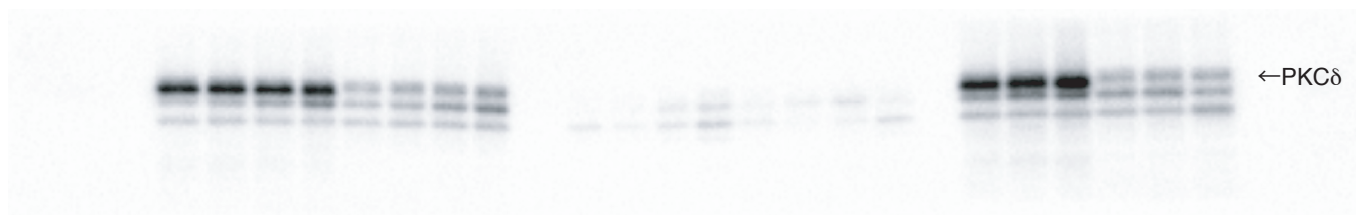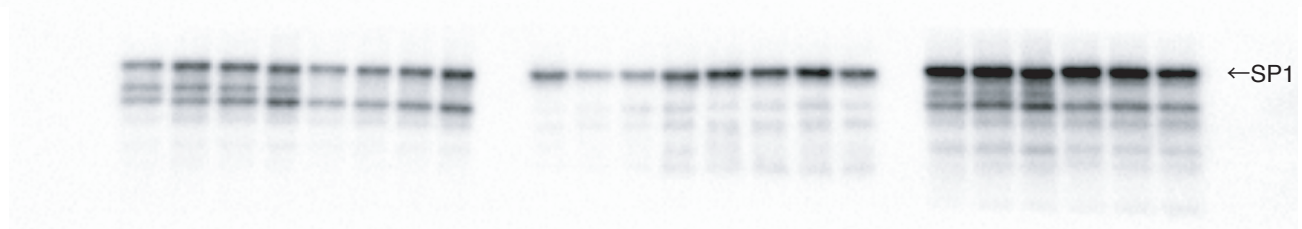

Supplement: Figure 5—source data 5. [file elife-72116-fig5-data5.pdf]

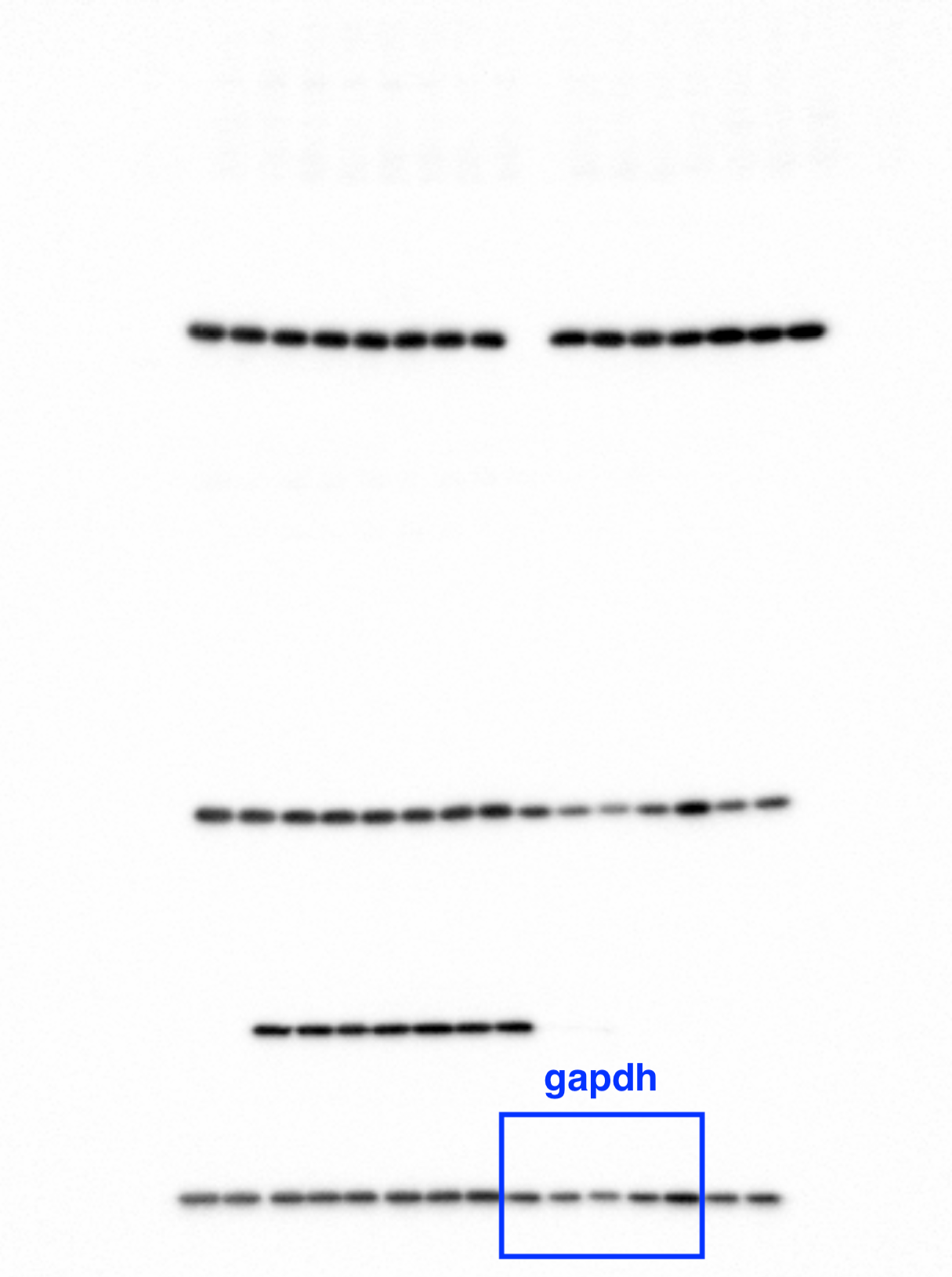

Supplement: Figure 5—figure supplement 1—source data 1. [file elife-72116-fig5-figsupp1-data1.tif]

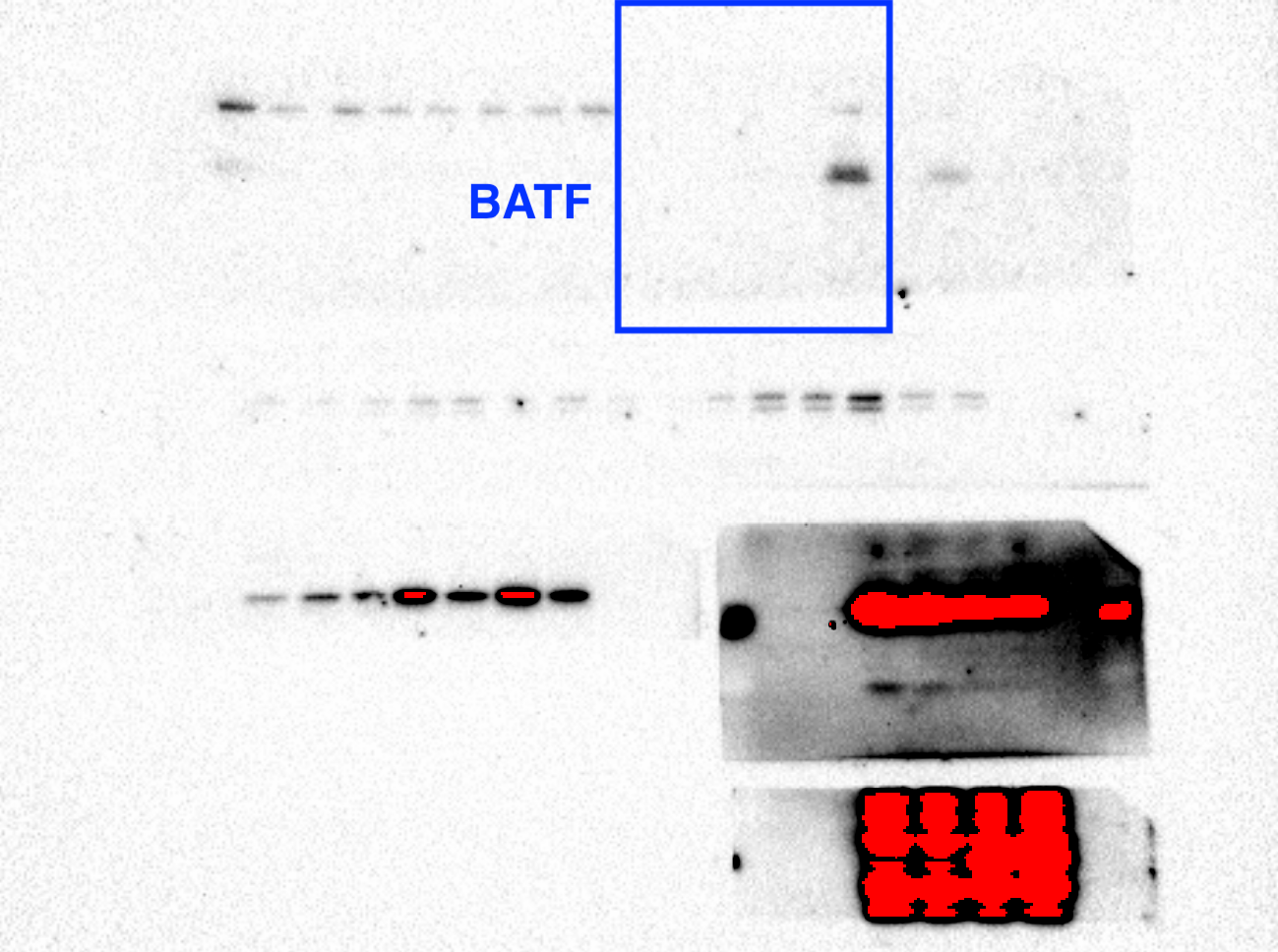

Supplement: Figure 5—figure supplement 1—source data 2. [file elife-72116-fig5-figsupp1-data2.tif]

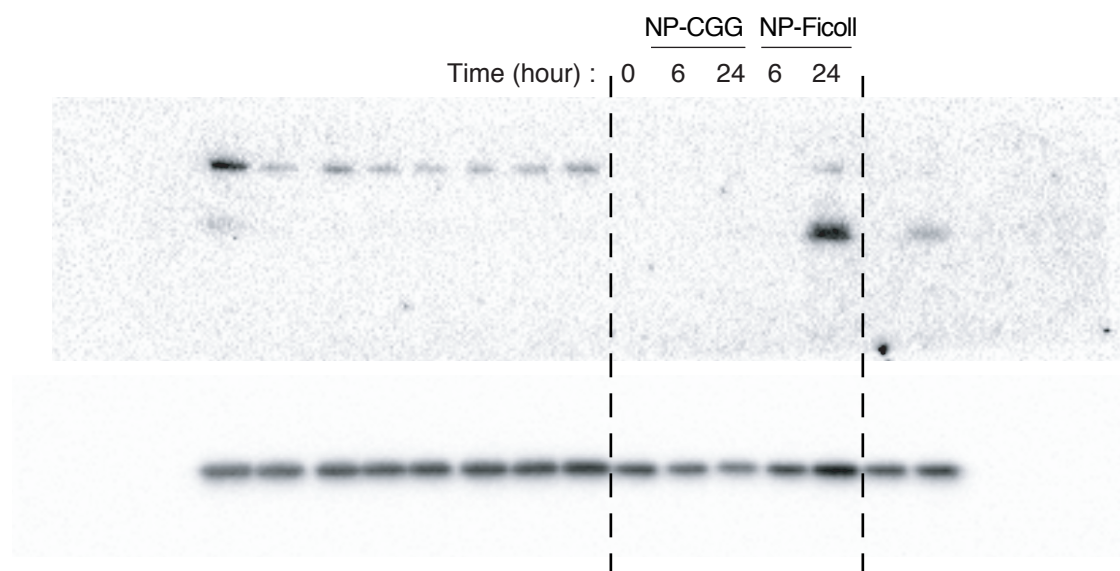

Supplement: Figure 5—figure supplement 1—source data 3. [file elife-72116-fig5-figsupp1-data3.pdf]

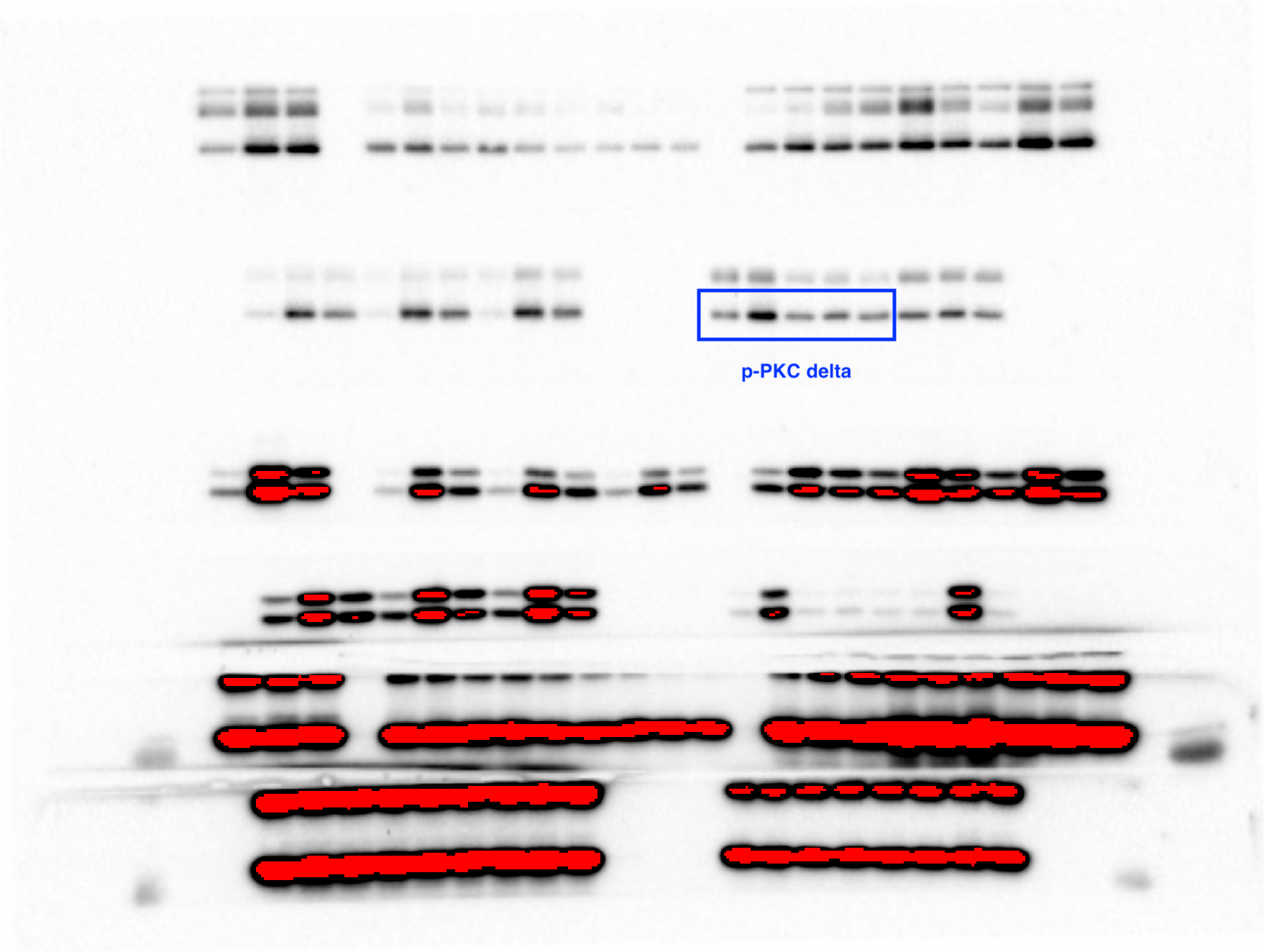

Supplement: Figure 5—figure supplement 1—source data 4. [file elife-72116-fig5-figsupp1-data4.tif]

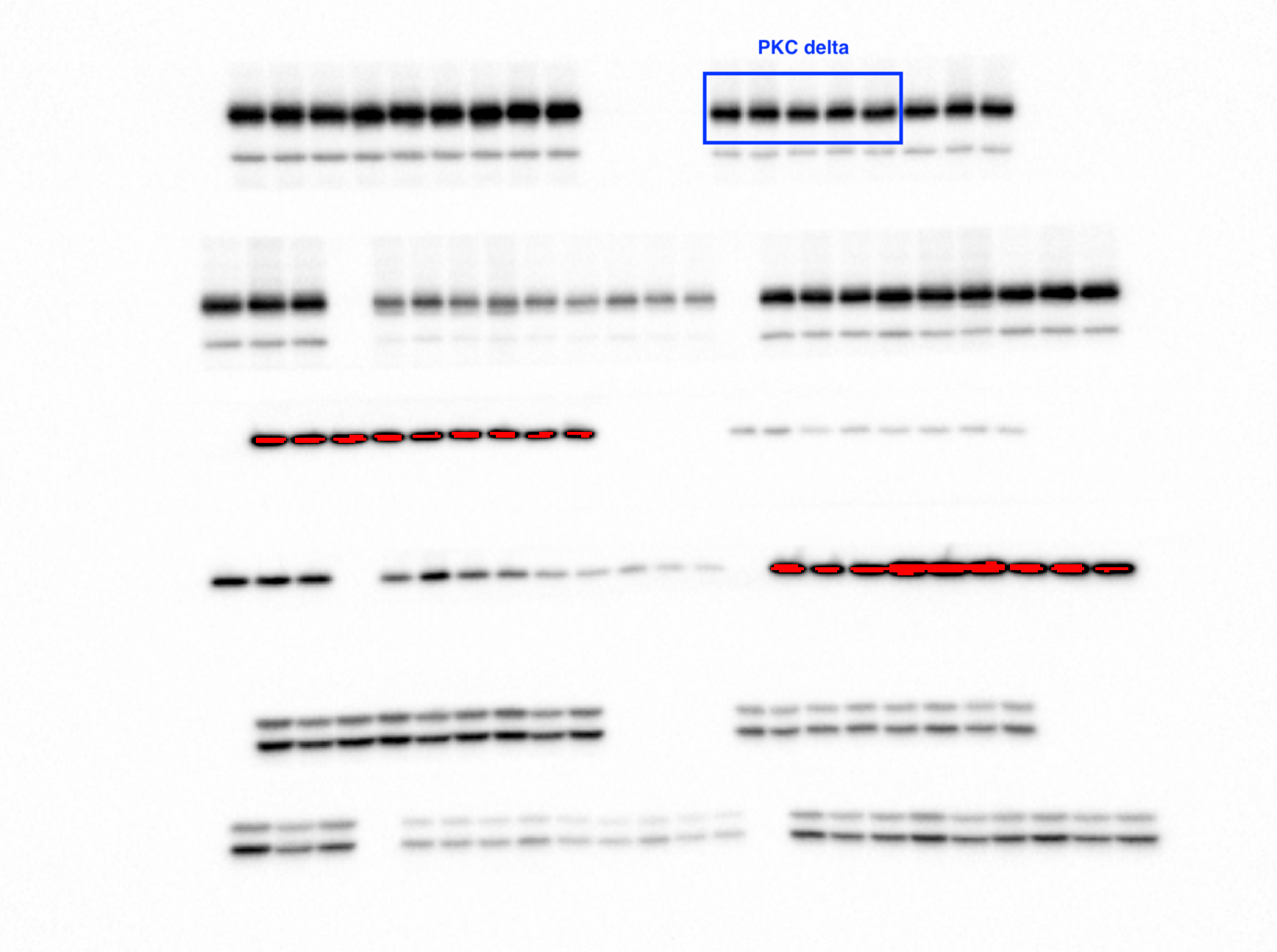

Supplement: Figure 5—figure supplement 1—source data 5. [file elife-72116-fig5-figsupp1-data5.tif]

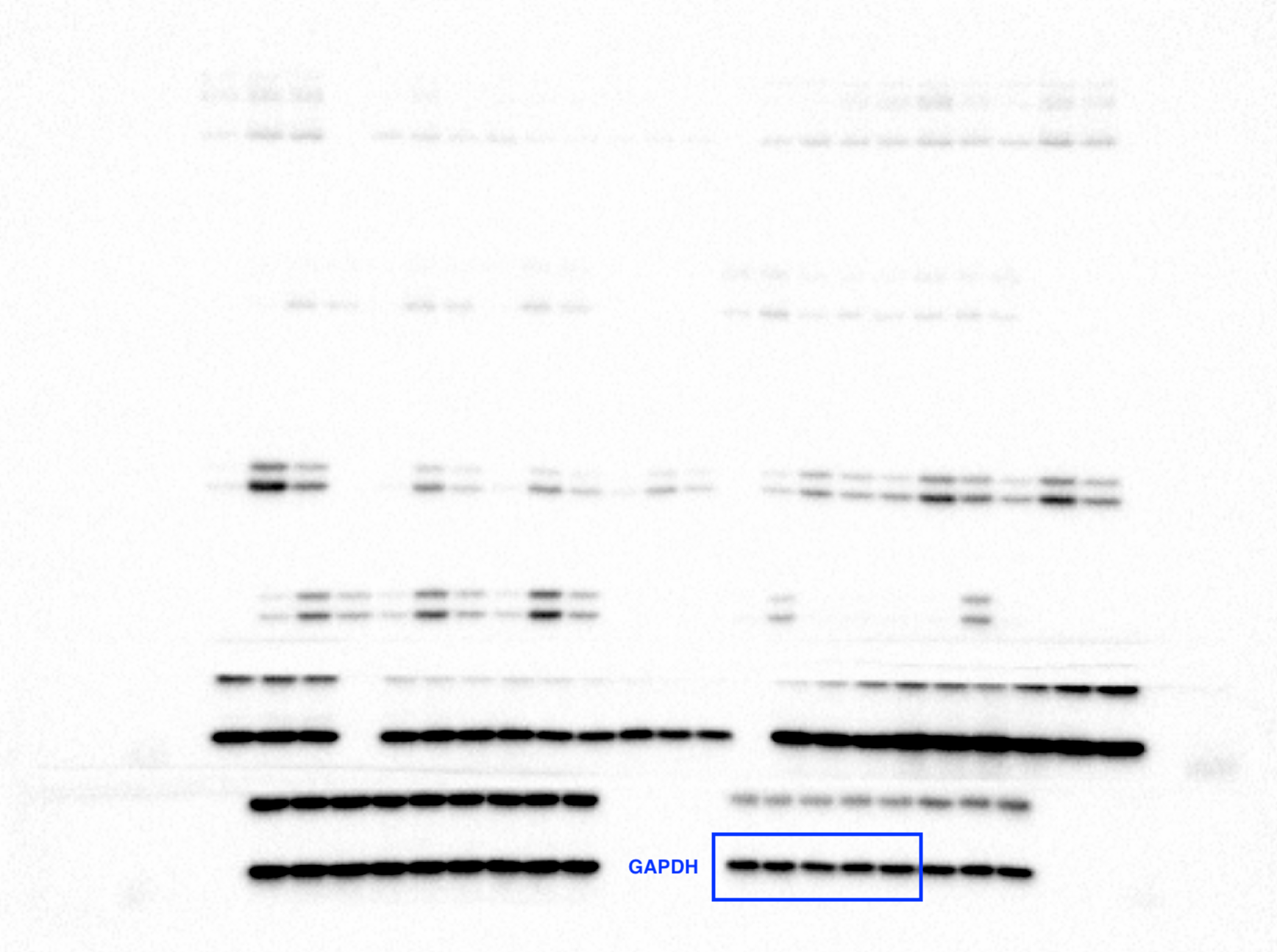

Supplement: Figure 5—figure supplement 1—source data 6. [file elife-72116-fig5-figsupp1-data6.tif]

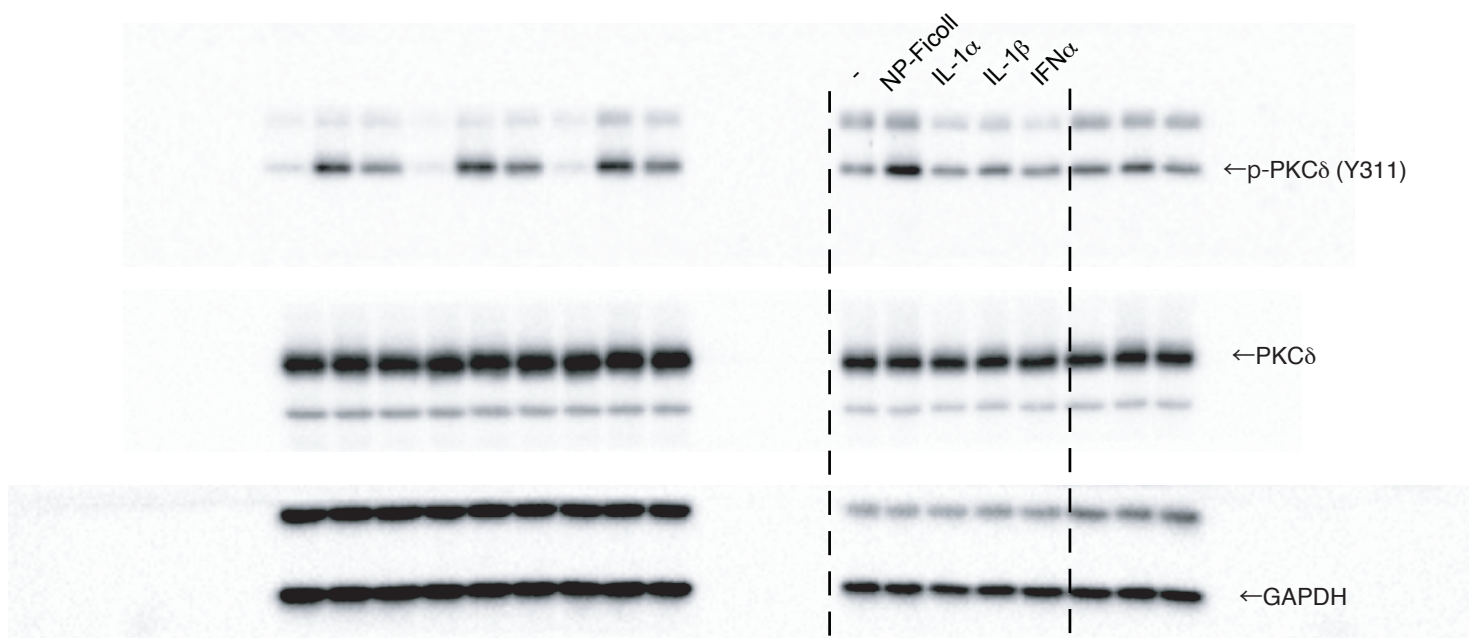

Supplement: Figure 5—figure supplement 1—source data 7. [file elife-72116-fig5-figsupp1-data7.pdf]

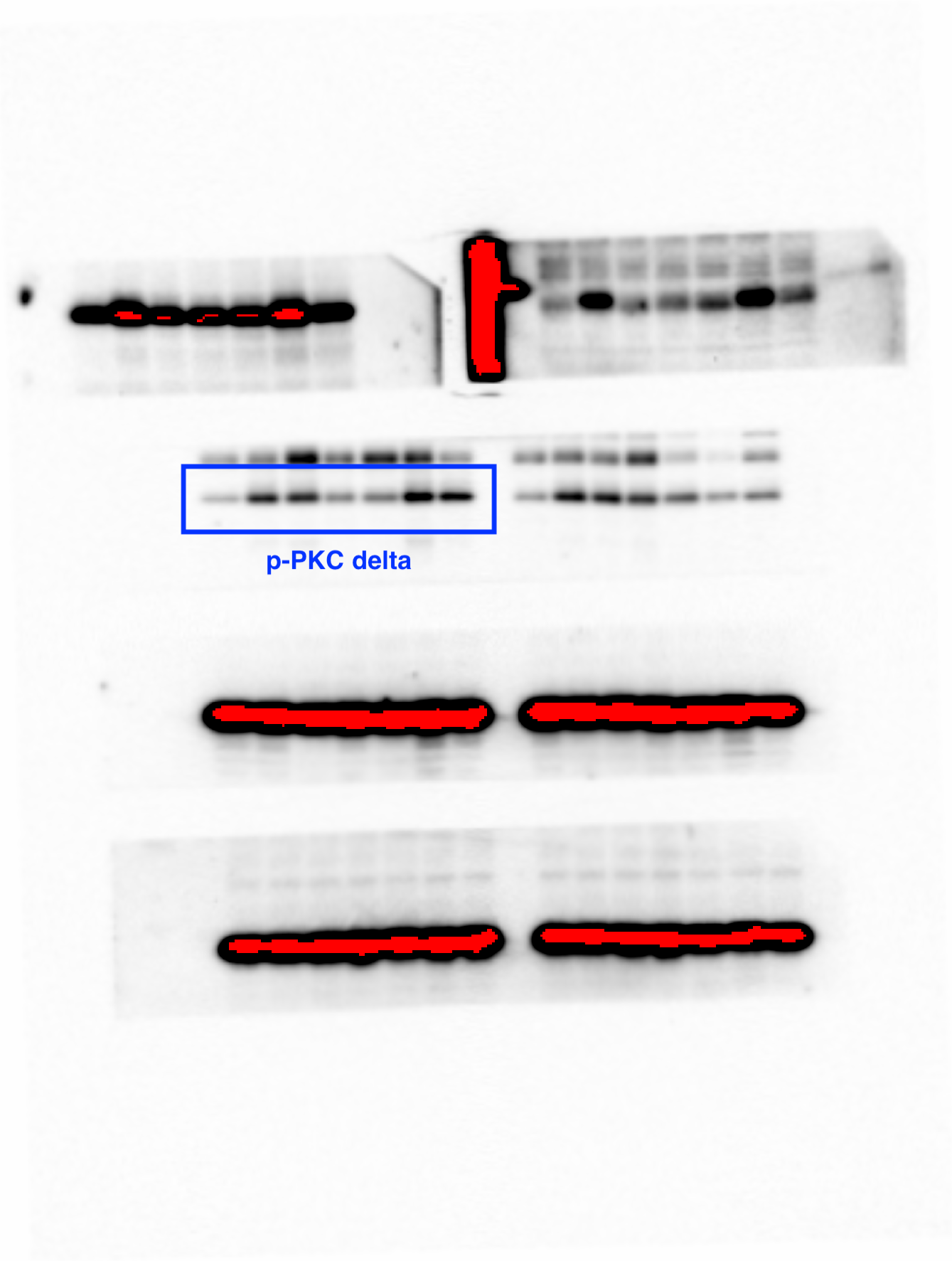

Supplement: Figure 5—figure supplement 1—source data 8. [file elife-72116-fig5-figsupp1-data8.tif]

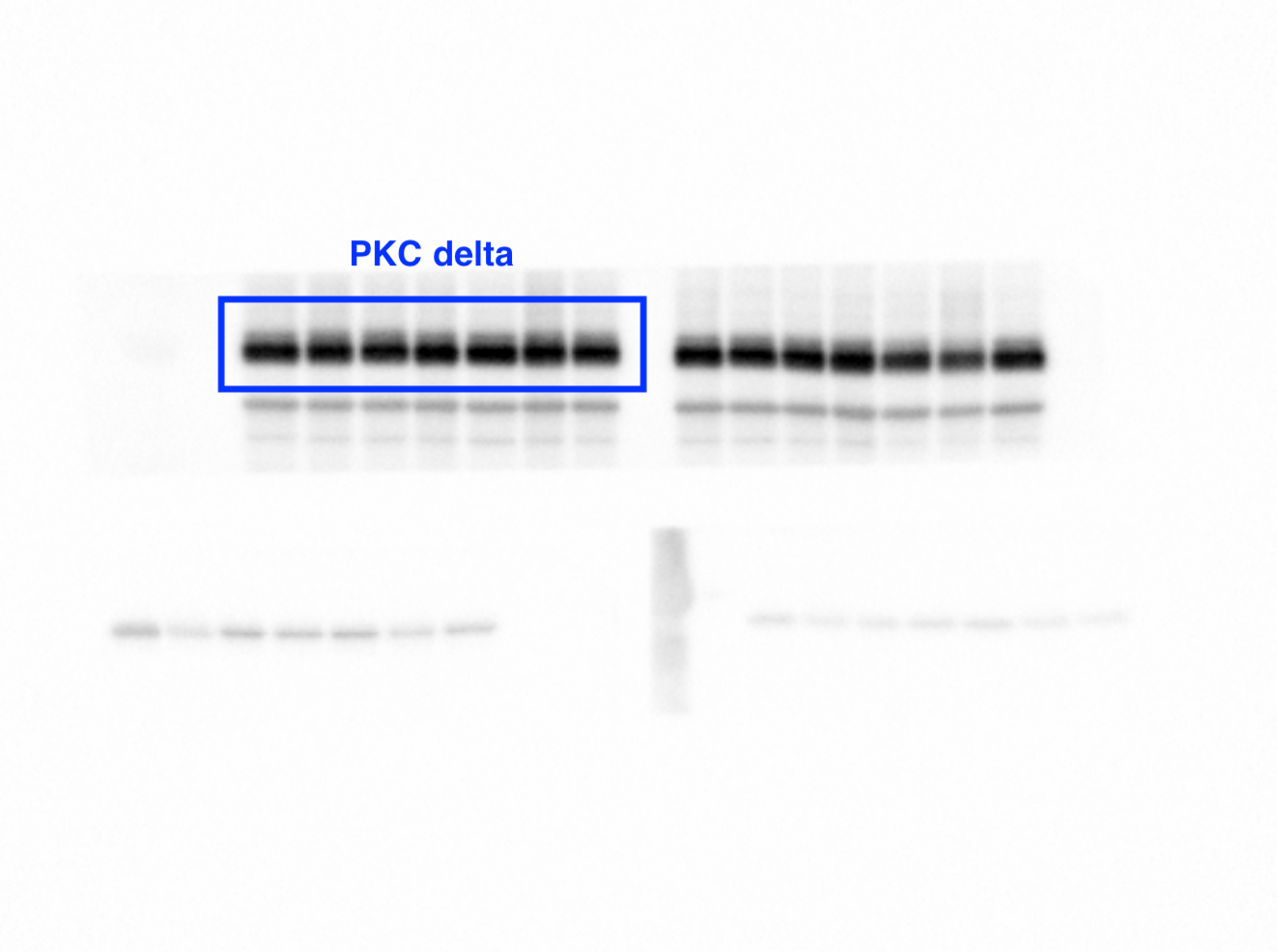

Supplement: Figure 5—figure supplement 1—source data 9. [file elife-72116-fig5-figsupp1-data9.tif]

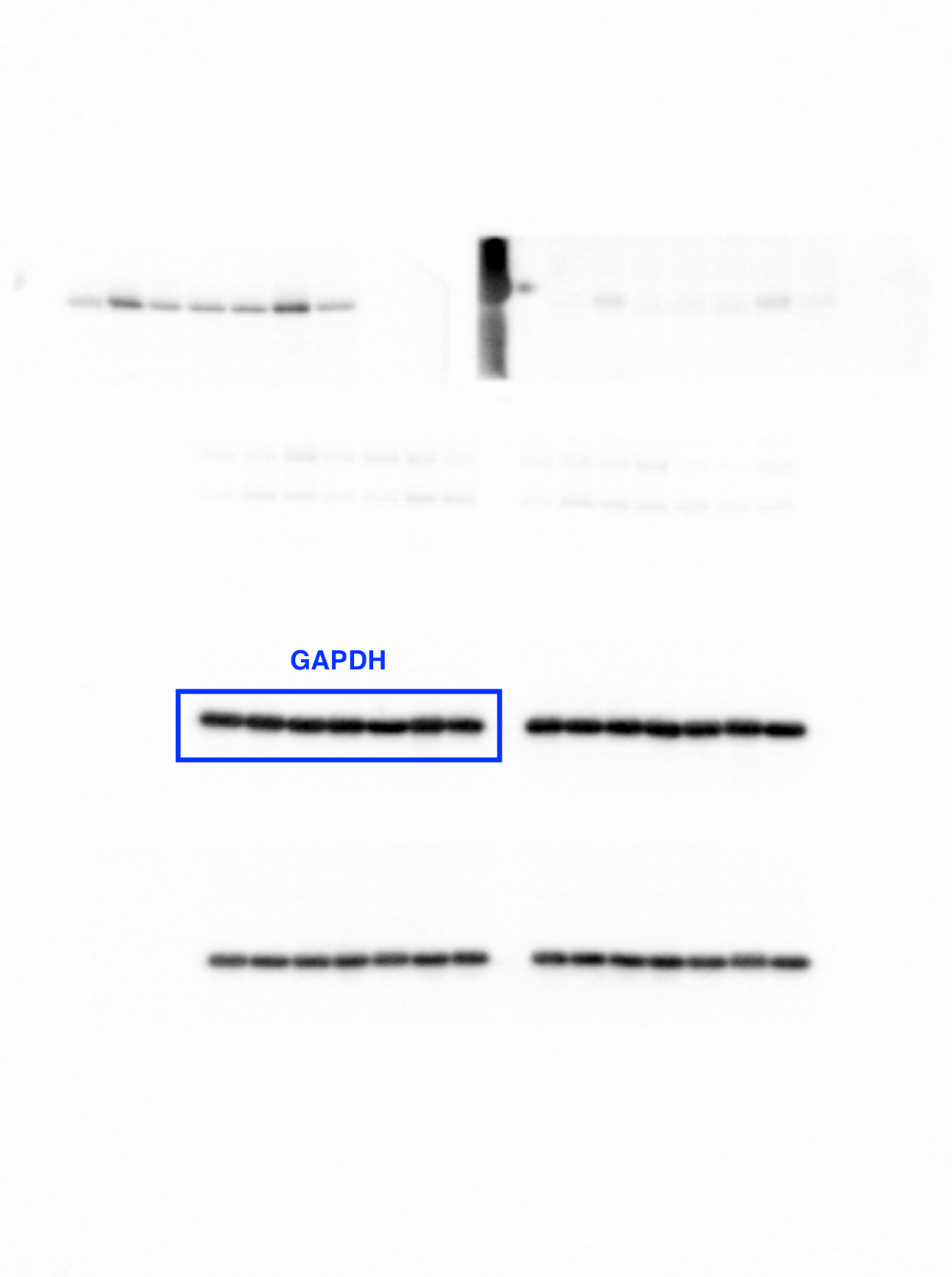

Supplement: Figure 5—figure supplement 1—source data 10. [file elife-72116-fig5-figsupp1-data10.tif]

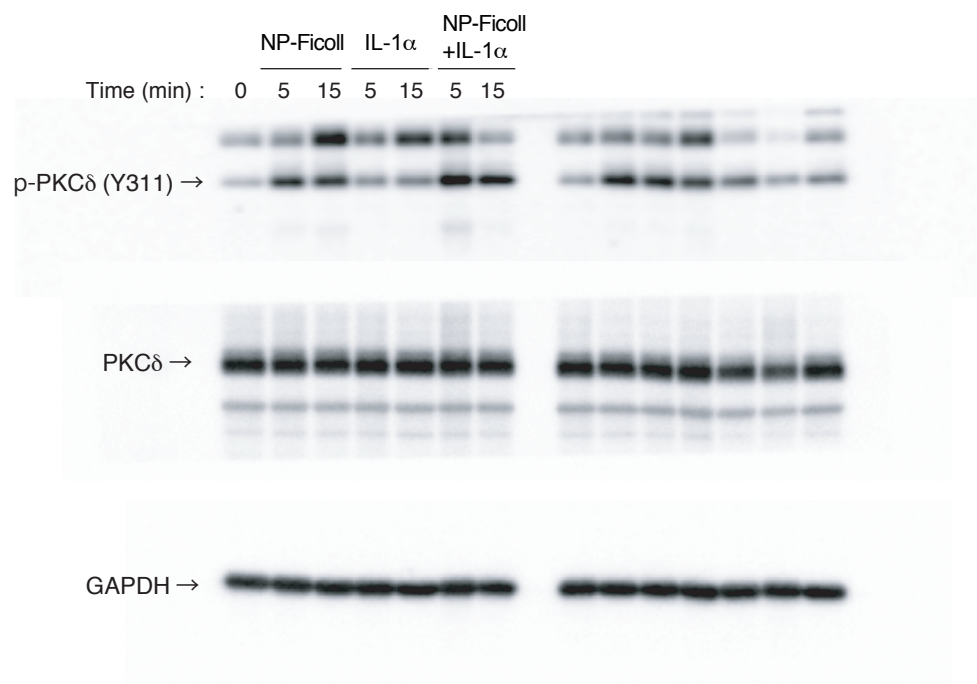

Supplement: Figure 5—figure supplement 1—source data 11. [file elife-72116-fig5-figsupp1-data11.pdf]
